# Fumiquinazolines F and G from the Fungus *Penicillium thymicola* Demonstrates Anticancer Efficacy Against Triple-Negative Breast Cancer MDA-MB-231 Cells by Inhibiting Epithelial–Mesenchymal Transition

**DOI:** 10.3390/ijms26157582

**Published:** 2025-08-05

**Authors:** Gleb K. Rystsov, Tatiana V. Antipova, Zhanna V. Renfeld, Lidiya S. Pilguy, Michael G. Shlyapnikov, Mikhail B. Vainshtein, Igor E. Granovsky, Marina Y. Zemskova

**Affiliations:** 1 G.K. Skryabin Institute of Biochemistry and Physiology of Microorganisms, Pushchino Scientific Center for Biological Research of the Russian Academy of Sciences, Pushchino 142290, Russia; gleb.8.ristsoff@gmail.com (G.K.R.); tatantip@rambler.ru (T.V.A.); zhanna.renfeld@yandex.ru (Z.V.R.); lida-vasileva-94@mail.ru (L.S.P.); shlyapnikov@ibpm.pushchino.ru (M.G.S.); vain@pbcras.ru (M.B.V.); granovsky@pbcras.ru (I.E.G.); 2 All-Russian Institute of Plant Protection, Pushkin, Saint-Petersburg 196608, Russia

**Keywords:** *P. thymicola*, quinoline alkaloids, fumiquinazolines F and G, MDA-MB-231 triple-negative breast cancer cells, epithelial–mesenchymal transition, anticancer activity, CD44

## Abstract

The secondary metabolites of the fungus *Penicillium thymicola*, fumiquinazolines F and G, have antibacterial and antifungal characteristics; however, their potential anti-tumor action against human cancer cells remains unknown. The goal of our study was to determine the biological efficacy of fumiquinazolines F and G on breast and prostate cancer cells. Cancer cell proliferation and migration were monitored in real time using xCELLigence technology and flow cytometry. Alterations in mRNA and protein expression were assessed by RT-qPCR, ELISA, and Western blotting. Our data indicate that fumiquinazolines F and G are more effective in inhibiting breast cancer cell proliferation than prostate cancer cells. Fumiquinazoline F is active against both hormone-dependent epithelial MCF-7 (IC_50_ 48 μM) and hormone-resistant triple-negative mesenchymal MDA-MB-231 breast cancer cells (IC_50_ 54.1 μM). The metabolite has low cytotoxicity but slows cell cycle progression. In fumiquinazoline F-treated MDA-MB-231 cells, the levels of proteins implicated in epithelial–mesenchymal transition (EMT) (such as E-cadherin, vimentin, and CD44) fluctuate, resulting in a decrease in cell migratory rate and adhesion to a hyaluronic acid-coated substrate. Thus, fumiquinazolines F and G exhibit anticancer activity by inhibiting EMT, cell proliferation, and migration, hence reverting malignant cells to a less pathogenic phenotype. The compound’s multi-target anticancer profile underscores its potential for further exploration of novel EMT-regulating pathways.

## 1. Introduction

Approximately 75% of low-molecular compounds employed in clinical oncology are not synthetic, but rather are natural molecules retrieved from diverse sources. Among these, 49% are secondary metabolites originating from fungi or bacteria, or their direct derivatives [1,2]. Secondary metabolites secreted by fungi of the genus *Penicillium* are promising sources of new chemical compounds with diverse biological activity [3,4]. One of the groups of compounds produced by these fungi is alkaloids, derivatives of quinoline. To date, over 240 representatives of the group, possessing various biological properties (antimalarial, anti-inflammatory, cardiovascular, antifungal, antitumor, etc.—at least 40 types of biological activity can be distinguished) have been identified. Ninety compounds, approximately 36% of the total identified alkaloids, exhibit anticancer activity, with around forty-five of these also possessing anti-inflammatory, antiviral, antifungal, or antibacterial characteristics [5,6].

One of the subgroups of quinoline alkaloids is fumiquinazolines-compounds containing an indole group and a pyrazino[2,1-b]quinazoline-3,6-dione core synthesized by the cell from anthranilic acid, tryptophan, and alanine [7,8]. Since their discovery in 1992, twenty-three fumiquinazolines found in nature have been isolated and characterized. A moderate cytotoxic activity to the murine lymphocytic leukemia line P-388 has been described for fumiquinazolines A-G (the highest for fumiquinazoline A, IC_50_ = 6.1 µg/mL; the lowest for fumiquinazoline E, IC_50_ = 52 µg/mL) [9]. Fumiquinazoline J exposes the anti-cancer activity against a broad range of tumor cell lines of mouse and human origin, including human ovarian therapy-sensitive cancer cells A2780sens (IC_50_ = 18.5 µM) and cisplatin-resistant subclone A2780CisR (IC_50_ = 38.8 µM) [10,11].

Fumiquinazolines F and G, which are released by the fungus *Penicillium,* are stereoisomers with a molecular weight of 358.4 g/mol (Figure S1) [12]. They have antifungal activities [13,14], but evidence for their possible cytotoxic effect against tumor cells is limited. As demonstrated, these metabolites are cytotoxic to murine P-388 cells and can reduce the proliferation of tsFT210 cells derived from malignant neoplasms of the mouse mammary gland [10]. However, the biological activity of these fumiquinazolines on human tumor cells of various etiologies and the mechanisms of their action remain unclear. We investigated whether fumiquinazolines F and G could suppress tumor cell growth using human cell lines derived from breast and prostate cancers. Our findings indicate that these compounds suppress the proliferation of cancer cells and the migration of triple-negative breast cancer cells MDA-MB-231. Retardation of cell migration is more likely associated with fumiquinazoline-induced alterations in the level of proteins involved in epithelial–mesenchymal transition (EMT), implying a partial reversal of the cancer cell state to a less pathological phenotype. This is the first study to show that fumiquinazolines F and G have anticancer activity against breast cancer cells of variable aggressiveness, specifically highly aggressive mesenchymal MDA-MB-231 cells.

## 2. Results

### 2.1. Fumiquinazolines Inhibit the Growth of Breast and Prostate Cancer Cells

We initially investigated whether fumiquinazolines F and G could inhibit tumor cell growth. Several human breast and prostate cancer cell lines were employed to mimic tumors with varying levels of aggressiveness. Breast cancer cell line MCF-7 belongs to the luminal A subtype, the least aggressive epithelial-like cell line, which retains some characteristics of differentiated mammary epithelium [15,16]. These cells express estrogen receptors (ER), as well as progesterone receptors (PR), and are used as a model for the treatment of ER/PR+ breast cancer with anti-estrogen drugs. BT474, commonly known as the luminal B subtype, is an epithelial-like cell line derived from human invasive ductal carcinoma of the breast. These cells are shown to be ER- and PR-positive; however, they have overexpression of HER2, which is associated with resistance to hormone therapy [17,18]. MDA-MB-231, which belongs to the basal-like subtype, is a highly aggressive, invasive, poorly differentiated triple-negative breast cancer (TNBC) cell line due to its lack of ER, PR, and HER2, and is known for its resistance to anti-cancer therapies [19,20].

Prostate cancer research typically relies on existing model cell lines such as LNCaP and PC3, which are known to be, respectively, lowly and highly metastatic. LNCaP cells express both androgen receptor (AR) and prostate-specific antigen (PSA), and their growth is inhibited by androgen withdrawal [21,22]. PC3 cells do not express AR and PSA and are used as a model of prostate cancer resistant to androgen-deprivation therapy [23,24].

**Figure 1:**
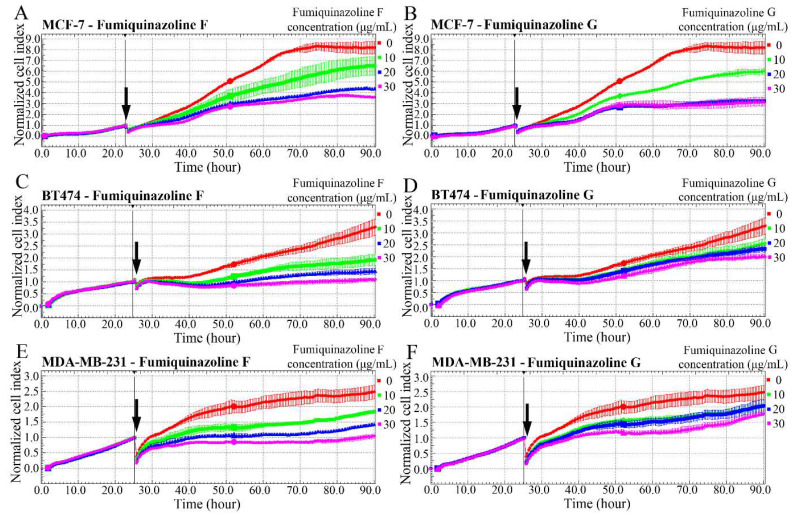
Fumiquinazolines F and G inhibit breast cancer cell proliferation. The xCELLigence technology was used to monitor real-time growth of breast cancer cell lines MCF7 (**A**,**B**), BT474 (**C**,**D**), and MDA-MB-231 (**E**,**F**) treated with fumiquinazolines F (**A**,**C**,**E**) or G (**B**,**D**,**F**) at dosages of 0, 10, 20, and 30 μg/mL. The *X*-axis depicts treatment time, while the *Y*-axis represents cell index values, which are proportional to cell confluency. The black arrows in the graphs indicate the time of addition of fumiquinazolines to the cell cultures. A representative experiment from three independent experiments is provided.

The xCELLigence technology was employed to examine alterations in cell growth rates caused by fumiquinazolines F and G. This approach allows measuring cell growth in a real-time manner according to the duration of treatment and concentration of the substances. As shown in Figure 1, fumiquinazolines F and G inhibited the growth of MCF-7 (Figure 1A,B), BT-474 (Figure 1C,D), and MDA-MB-231 (Figure 1E,F) breast cancer cells. The effects were noticed 10 h after the addition of the substances, while the inhibition of cell growth for prostate cancer cells LNCap (Figure S2A,B) began 16 h after drug administration. PC3 cells responded poorly to fumiquinazolines F and G treatment (Figure S2C,D). The half-maximal inhibitory concentration of metabolites was calculated relative to the untreated control (see Table 1).

IC_50_ rates indicate that both fumiquinazolines limit cell growth at micromolar concentrations, with hormone-sensitive prostate LNCaP and breast MCF-7 cancer cells being more vulnerable to the treatment. Unlike AR^+^ LNCaP, AR^−^ prostate cancer cells PC3, which also have a null mutation in the *TP53* gene and a loss of PTEN, are resistant to fumiquinazoline-induced growth suppression. However, fumiquinazoline F can inhibit the proliferation of hormone therapy-resistant breast cancer cells BT-474 and MDA-MB-231, suggesting its potency for the treatment of breast cancer, and fumiquinazoline F has a more inhibitory effect than its stereoisomer fumiquinazoline G (Table 1). Triple-negative MDA-MB-231 cells are different from other breast cancer cells, possessing the greatest aggressiveness and multidrug resistance [25,26]. Therefore, the inhibitory activity of fumiquinazolines on the multiplication and spreading of these cells is of particular interest.

### 2.2. Cytotoxic and Cytostatic Effects of Fumiquinazoline F on MDA-MB-231 Cells

**Figure 2:**
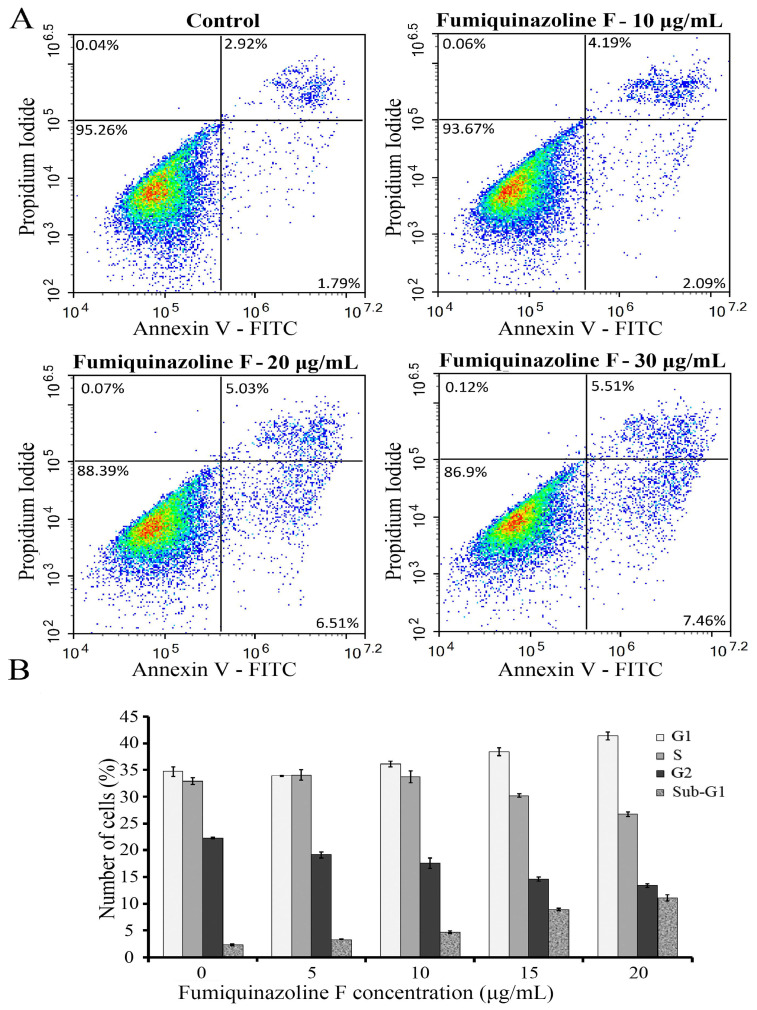
Fumiquinazoline F does not cause extensive cell death but rather inhibits cell proliferation. (**A**) MDA-MB-231 cells were cultured for 48 h in either the absence or presence of fumiquinazoline F (0–30 μg/mL). The cytotoxicity in cell cultures was next assessed by annexin V (AnV)-propidium iodate (PI) staining, followed by flow cytometry analysis. Cell distribution in untreated (control) and fumiquinazoline-treated cell populations: the lower left panel shows non-stained AnV-/PI- alive cells, the upper left panel indicates AnV-/PI+ necrotic cells, the lower right panel shows AnV+/PI- early apoptotic cells, and the upper right panel points to AnV+/PI+ late apoptotic cells. The percentages of unstained and stained cells are shown in each panel. Different colors reflect the number of cells. Intensive red and yellow show that a majority of cells are annexin V and PI negative and cell death is absent. (**B**) Cell cycle analysis of MDA-MB-231 cells incubated for 24 h in the absence or presence of fumiquinazoline F (0–20 μg/mL). The histogram displays the number of cells (*Y*-axis) at each cell cycle stage at respective fumiquinazoline F concentrations (*X*-axis). The results are shown as the mean ± SD of four independent experiments.

Because suppression of cell growth could indicate either the induction of cell death or a slowing of cell division rate, or both, we assessed the apoptosis level and conducted a cell cycle analysis. The annexin V-FITC/propidium iodide (PI) staining, followed by flow cytometric analysis of MDA-MB-231 cells treated with various doses of fumiquinazoline F, was employed. As shown in Figure 2, apoptotic (annexin V-FITC-positive) and necrotic (PI-positive) cells occur in the fumiquinazoline F-treated cell population, and their proportion rises with increasing concentration of the substance. However, treatment with a fumiquinazoline F concentration of 10 μg/mL (26 μM) resulted in a slight increase in the number of late apoptotic cells (from 2.92% to 4.19%). Also, after an increase in the fumiquinazoline F concentration to 30 μg/mL (78 μM), only a moderate increase in the number of early apoptotic cells by 4.2 times (from 1.79% to 7.46%) and late apoptotic cells by 2.7 times (from 2.92% to 5.51%) was observed (Figure 2A). In addition, a treatment with fumiquinazoline F at a dose of 30 μg/mL (78 μM) did not significantly increase the number of necrotic cells. Therefore, fumiquinazoline F, even within a concentration range greater than the IC_50_ value, had a weak cytotoxicity, which caused MDA-MB-231 cell death via the apoptotic rather than necrotic pathway. After a treatment with various amounts of fumiquinazoline F, flow cytometry was employed to look at how MDA-MB-231 cells stained with a DNA-binding dye spread out across the different stages of the cell cycle (Figure 2B). In addition to detecting cell populations at different stages of the cell cycle, this assay enables the identification of apoptotic cells with DNA content less than 2N (sub-G1). As shown in Figure 2B, the fumiquinazoline F treatment resulted in a concentration-dependent increase in the number of cells in the G1 phase and a decrease in the cell population in the S and G2 phases. An accumulation of apoptotic cells (sub-G1) was also observed. These data indicate that, in addition to cytotoxicity, fumiquinazoline F has cytostatic capacities by slowing the MDA-MB-231 cell proliferation and leading to the accumulation of cells in the G1 stage of the cell cycle.

### 2.3. Fumiquinazoline F Inhibits MDA-MB-231 Cell Migration

**Figure 3:**
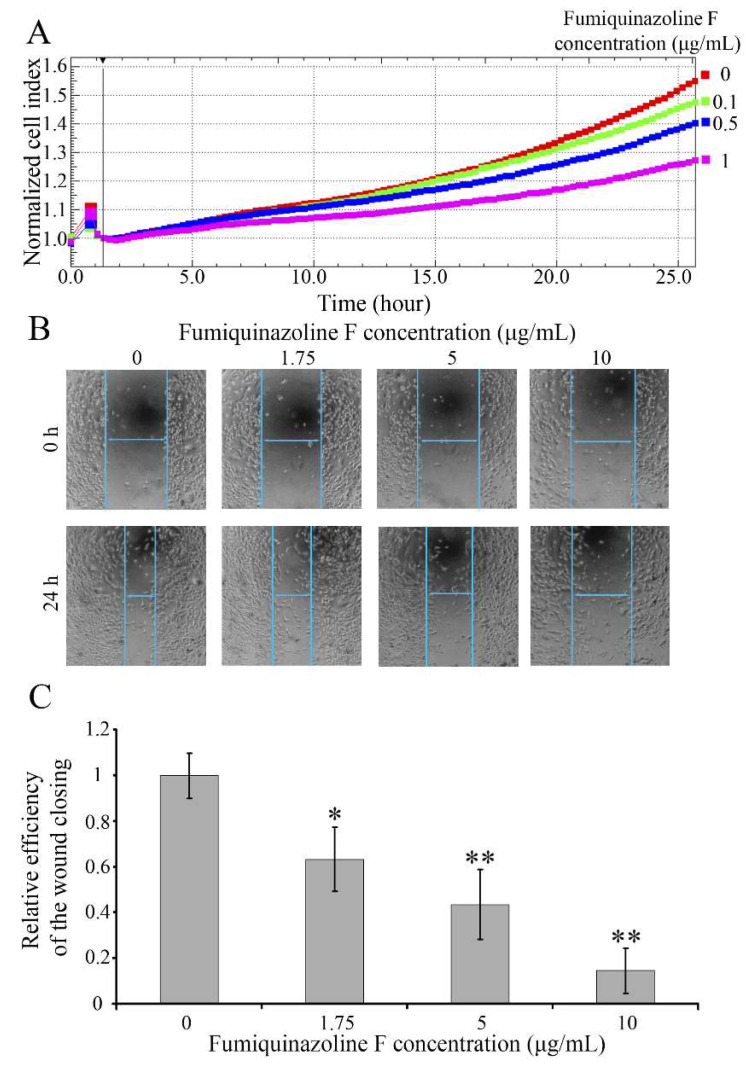
Fumiquinazoline F inhibits MDA-MB-231 cell migration. (**A**) The migratory dynamics of MDA-MB-231 cells via a porous membrane towards a fetal bovine serum gradient were observed in real time using xCelligence technology. The *X*-axis represents cell migration time. The *Y*-axis shows cell index change, indicating the number of migrated cells. (**B**) The migration of MDA-MB-231 cells was evaluated using a scratch tightening assay. The top row shows micrographs of the cell-free area at the start (0 h), while the bottom row shows the same area after 24 h of incubation with different concentrations of fumiquinazoline F (0–10 μg/mL). The lines represent the area in which migration intensity was assessed. (**C**) The histogram of the migration rate of MDA-MB-231 cells computed from the results of three independent experiments. The *X*-axis represents the concentration of fumiquinazoline F. The *Y*-axis depicts the relative efficiency of cell migration into the scratch’s cell-free space. The results are shown as the mean ± SD, and *p* values were calculated according to Student’s *t*-test. *—*p* < 0.05; **—*p* < 0.01.

The potential of MDA-MB-231 cells to form metastases is functionally expressed in their increased ability to migrate and can be estimated as the cell movement through a porous membrane along a chemoattractant gradient, with subsequent assessment of the number of migrated cells. To investigate how MDA-MB-231 cells treated with fumiquinazoline F migrated, an electrode-integrated Boyden chamber (CIM plates) and xCELLigence technology were used to track cell migration in real time. As shown in Figure 3A, fumiquinazoline F inhibits cell migration through a porous membrane along a chemoattractant gradient in a concentration-dependent manner. The half-inhibitory concentration IC_50_ of 1.75 μg/mL (4.54 μM) was determined by measuring the kinetics of tumor cell movement.

The inhibition of cell migration induced by fumiquinazoline F was also confirmed by the scratch tightening assay. As demonstrated in Figure 3B,C, suppression of MDA-MB-231 cell migration increases with increasing fumiquinazoline F concentration, and, as it rises, the areas unfilled with migrating cells become larger. Furthermore, it should be noted that the IC_50_ required to inhibit MDA-MB-231 cell migration is much less than that obtained for cell growth suppression, suggesting that fumiquinazoline F could have a greater potential when used as an anti-metastatic drug rather than an inhibitor of tumor cell growth.

### 2.4. Fumiquinazolines F and G Increase E-Cadherin and Decrease Vimentin Protein Levels in MDA-MB-231 Cells

**Figure 4:**
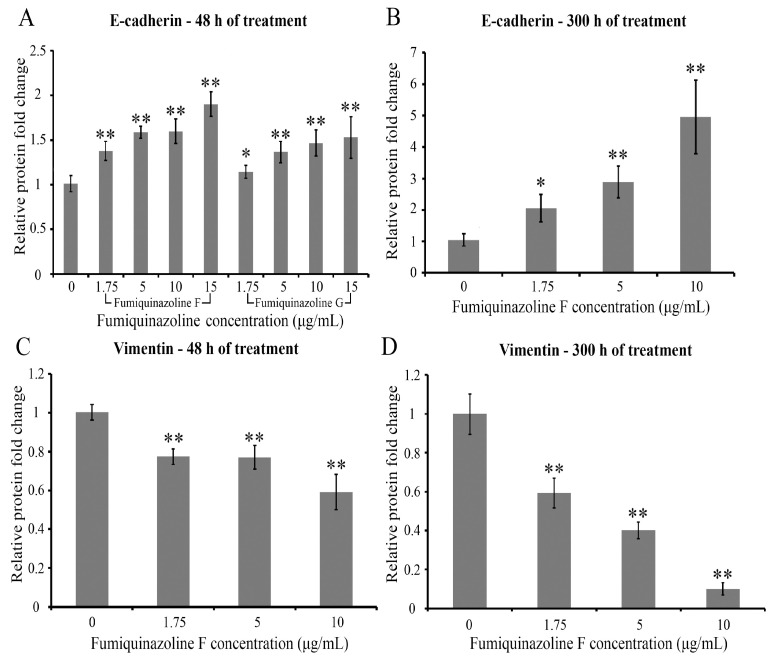
Fumiquinazolines F and G increase E-cadherin and decrease vimentin levels in MDA-MB-231 cells. Histograms of ELISA results for MDA-MB-231 lysates following cell incubation with fumiquinazolines F and G for different durations. The treatment causes concentration-dependent alterations in E-cadherin (**A**,**B**) and vimentin (**C**,**D**) levels. The effect is more significant after 300 h of fumiquinazoline administration (**B**,**D**) vs. 48 h of treatment (**A**,**C**). The *X*-axis represents the concentration of fumiquinazoline F; the *Y*-axis represents the relative amount of protein. The results are shown as the mean ± SD of three independent experiments, measured in triplicate. *—*p* < 0.05; **—*p* < 0.01, *p* values were calculated according to the Student’s *t*-test.

MDA-MB-231 is a cell line that has undergone EMT [27,28,29]. This process converts epithelial cells to a mesenchymal phenotype, resulting in a greater migratory potential of cancer cells. Using real-time qPCR, we showed that, unlike epithelial breast cancer MCF-7 cells, MDA-MB-231 cells expressed high levels of mesenchymal markers such as vimentin and slug (SNAI2), but a low level of epithelial E-cadherin mRNA (Figure S3A). Because fumiquinazoline F inhibits migratory activity in MDA-MB-231 cells, we investigated whether fumiquinazoline F could alter the EMT phenotype of MDA-MB-231 cells. Real-time qPCR was performed to compare EMT marker expression in untreated control cells and MDA-MB-231 cells incubated for 48 h with fumiquinazoline F at doses ranging from 1.75 to 15 μg/mL (4.54 to 39 μM). No change was found in the mRNA levels of EMT marker proteins N-cadherin, E-cadherin, β-catenin, CD44, and vimentin or EMT-related transcription factors Slug (SNAI2), Snail (SNAI1), TWIST1, FOXC1, ZEB1, and ZEB2. These results suggest that fumiquinazoline F has no effect on the transcription of these genes. To investigate whether fumiquinazoline F alters the expression of EMT marker proteins, the enzyme-linked immunosorbent assay (ELISA) was employed. The levels of vimentin and E-cadherin were estimated in cell lysates of MDA-MB-231 cells treated with fumiquinazoline F at concentrations ranging from 1.75 μg/mL (IC_50_ for inhibition of cell migration) to 10 μg/mL. It was shown that incubation of MDA-MB-231 breast cancer cells in the presence of fumiquinazoline F resulted in a concentration-dependent increase in the level of E-cadherin (Figure 4A,B) and a decrease in the level of vimentin (Figure 4C,D). It is worth noting that, for both proteins, the observed effect became more robust with the duration of the treatment time (Figure 4B,D).

As a result, fumiquinazoline F alters the levels of vimentin and E-cadherin, indicating a shift of MDA-MB-231 cells toward an epithelial phenotype.

### 2.5. Fumiquinazoline F Reduces CD44 Protein Levels in MDA-MB-231 Mesenchymal Cells

**Figure 5:**
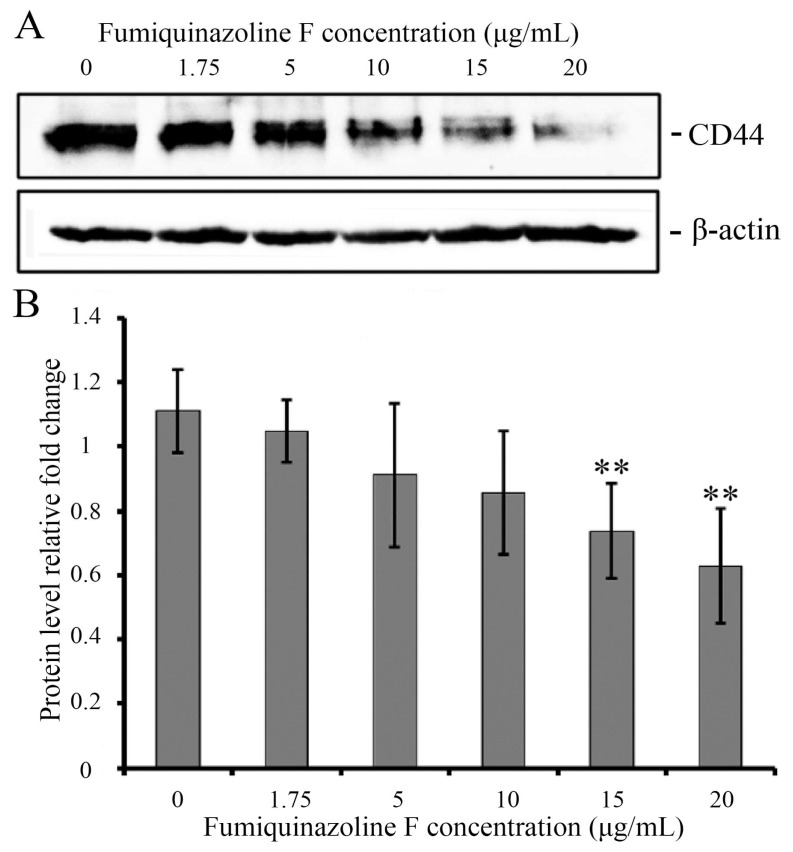
Fumiquinazoline F reduces the CD44 protein level in MDA-MB-231 mesenchymal cells. (**A**) Western blot analysis of cell lysates following a 48 h treatment of MDA-MB-231 cells with varying concentrations of fumiquinazoline F (0–20 μg/mL). β-Actin was used to control protein loading. (**B**) Histogram representing the densitometric analysis of Western blot results. The signal intensities were estimated using Image studio software version 5 (LI COR, Lincoln, NE, USA) and the CD44 protein level was normalized to β-actin. The *Y*-axis shows the relative amount of protein, while the *X*-axis represents the concentration of fumiquinazoline F. The results are shown as the mean ± SD of six independent experiments. **—*p* < 0.01, *p* values were calculated according to the Student’s *t*-test.

One of the mechanisms implementing cell migration is the interplay of cancer cells with the extracellular matrix. The integral cellular glycoprotein and hyaluronic acid receptor CD44 plays an important role in this process and is related to breast cancer malignancy [30,31,32]. CD44 is expressed in many isoforms, with the standard isoform being the most prevalent in MDA-MB-231 breast carcinoma cells [33,34,35]. As cells that have transitioned from an epithelial to a mesenchymal phenotype during EMT are known to express high levels of CD44 [36,37,38], we evaluated the expression of CD44 protein in epithelial MCF-7 and mesenchymal MDA-MB-231 cells. Western blot analysis revealed that the protein was nearly completely absent in MCF-7 but was present in significant concentrations in MDA-MB-231 cells (Figure S3B). Considering that fumiquinazoline F inhibits MDA-MB-231 cell migration, we hypothesized that the level of CD44 protein would be altered. Western blot analysis using CD44-specific antibodies revealed that fumiquinazoline F could cause a dose-dependent decrease in CD44 protein levels (Figure 5).

**Figure 6:**
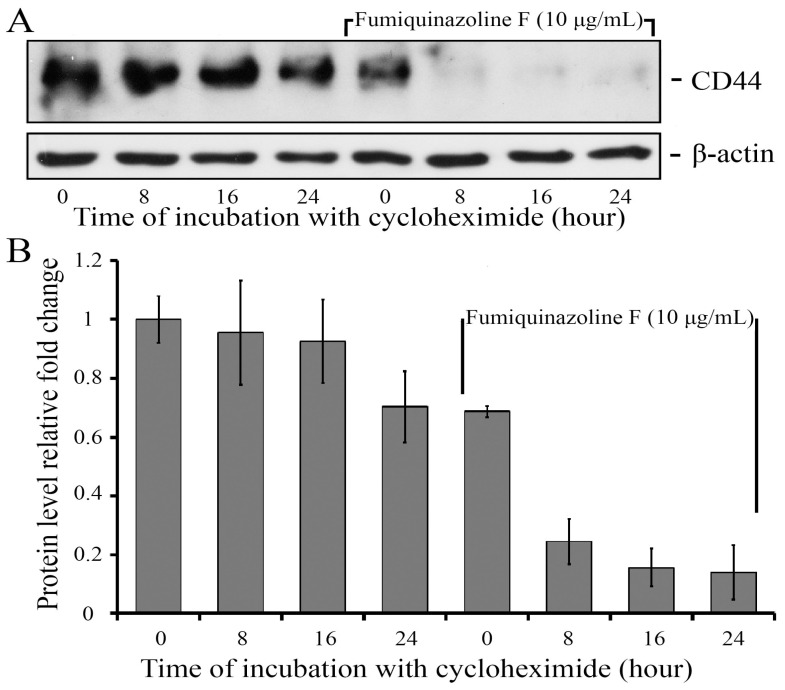
Fumiquinazoline F decreases the stability of CD44 protein in MDA-MB-231 mesenchymal cells. (**A**) Western blot analysis of MDA-MB-231 cell lysates treated with and without fumiquinazoline F (10 μg/mL) in the presence of cycloheximide (50 μg/mL) for the indicated times. β-Actin was employed as a protein loading control. (**B**) Histogram of densitometric analysis of the Western blot data. Protein levels were determined based on the image’s signal intensity using Image studio software version 5 (LI COR, Lincoln, NE, USA). The *Y*-axis represents the relative amount of CD44 protein normalized to β-actin. The *X*-axis shows the time of treatment with cycloheximide (50 μg/mL). The results are shown as the mean ± SD of three independent experiments.

As the amount of CD44 transcript did not change in MDA-MB-231 cells treated with fumiquinazoline F, we postulated that the observed decrease in CD44 protein was caused by a reduction in its stability. To test this hypothesis, the CD44 level in MDA-MB-231 cells was evaluated in the presence or absence of fumiquinazoline F while protein synthesis de novo was blocked by cycloheximide. The results show that in cycloheximide-treated MDA-MB-231 cells, the half-life of CD44 is more than 24 h, which is reduced to 8 h in the presence of the metabolite. Therefore, the decline in CD44 amount occurs faster than in cells treated with cycloheximide alone (Figure 6). These data suggest that fumiquinazoline F can affect CD44 protein stability.

### 2.6. Fumiquinazoline F Inhibits MDA-MB-231 Cell Adhesion to a Hyaluronic Acid-Coated Scaffold

**Figure 7:**
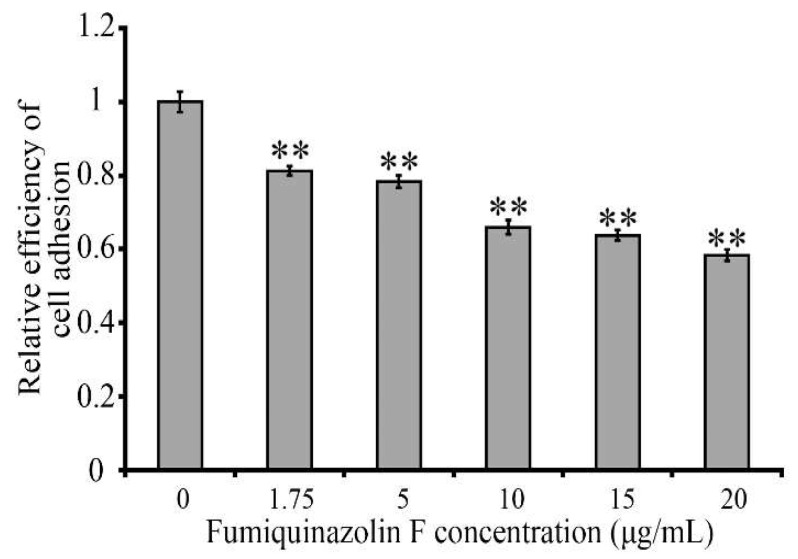
Fumiquinazoline F reduces MDA-MB-231 cells’ ability to attach to an HA-coated substrate. Histogram showing attachment test results for MDA-MB-231 cells treated for 48 h with varying concentrations of fumiquinazoline F (0–20 μg/mL). The *X*-axis displays the concentration of fumiquinazoline F, while the *Y*-axis shows the number of adhered cells. The results are presented as the mean ± SD of three independent experiments, with cell plating in quadruplicates for each treatment and measurement. **—*p* < 0.01; *p* values were computed using the Student’s *t*-test.

The main function of CD44 is to regulate cell migration by recognizing and binding to hyaluronic acid (HA) of the intercellular matrix. We expected that the drop in CD44 protein level induced by fumiquinazoline F would hinder MDA-MB-231 cell adhesion to an HA-coated substrate. Employing a cell attachment experiment, we determined that fumiquinazoline F treatment diminished the cells’ ability to adhere to a HA-coated substrate by a factor of 1.6 relative to the untreated control (Figure 7). We can presume that this is due to a decline in CD44 protein levels.

### 2.7. Fumiquinazoline F and Proteasome Inhibitor MG132 Combine Synergistically to Reduce CD44 Protein Level and Retard MDA-MB-231 Cell Growth

**Figure 8:**
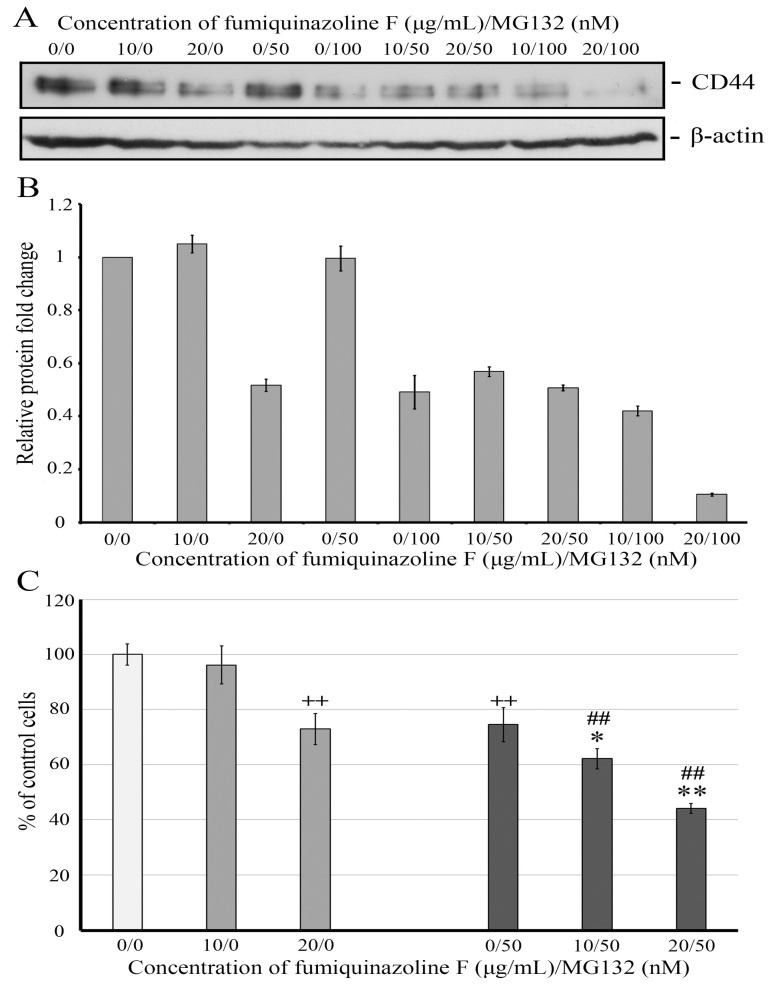
Fumiquinazoline F and MG132 reduce the amount of CD44 protein in MDA-MB-231 cells, and their combined treatment has a synergistic effect. (**A**) Western blot analysis of MDA-MB-231 cell lysates treated for 24 h either with fumiquinazoline F (10 and 20 μg/mL) or MG132 (50 and 100 nM) or their combination. β-Actin was used as a control for protein loading. (**B**) A histogram of densitometric analysis of Western blot data. The *Y*-axis shows the amount of protein relative to the CD44 level in untreated cells. The *X*-axis shows the concentrations of fumiquinazoline F, MG132, and their combination used in the treatment. The results are represented as the mean ± SD of three independent experiments. (**C**) Fumiquinazoline F increases the inhibitory effect of MG132 on MDA-MB-231 viability. Cells were treated for 24 h with fumiquinazoline F (10 and 20 μg/mL) with or without MG132 (50 nM). Following the incubation, cells were subjected to the crystal violet assay, as described in Materials and Methods. The results are shown as the mean ± SD of two independent experiments in which cell plating was repeated in eight wells for each treatment and measurement. ++—*p* < 0.01 compared to untreated cells. *—*p* < 0.05 and **—*p* < 0.01 compared to MG132 alone; ##—*p* < 0.01 compared to fumiquinazoline F alone.

The main mechanism of degradation of the receptor protein CD44 is its cleavage in lysosomes. However, MG132-induced proteasome blockade results in an increase in CD44 protein in epithelial cells such as T24T (invasive human transitional cell carcinoma of the bladder) [39], HuH-7 (human hepatocarcinoma), and PLC/PRF/5 (human hepatoma) [40], implying that, in these cells, proteosomes may play a role in the regulation of CD44 stability. Having found that fumiquinazoline F can diminish CD44 protein expression, we decided to examine whether the proteasome inhibitor MG132 might restore the CD44 level in MDA-MB-231 cells and rescue them from the effect of the metabolite. Western blot analysis of MDA-MB-231 cell lysates from untreated cells and those treated for 24 h with varying concentrations of the proteasome inhibitor MG132, fumiquinazoline F, or their combinations revealed that fumiquinazoline F alone at 10 μg/mL and MG132 alone at 0.25 μg/mL (50 nM) did not yield statistically significant alterations in CD44 levels. However, their combination resulted in a decrease in the CD44 amount, indicating a possible synergism of their interaction (Figure 8A,B). As expected, raising the fumiquinazoline F concentration to 20 μg/mL lowered the CD44 level twofold. Interestingly, increasing the concentration of MG132 to 0.5 μg/mL (100 nM) did not cause CD44 accumulation; instead, it decreased its amount by 2.2 times. The co-treatment of MDA-MB-231 cells with fumiquinazoline F at a concentration of 20 μg/mL and MG132 at 0.5 μg/mL (100 nM) resulted in a 4.62-fold reduction in CD44 levels, indicating an additive effect of the two agents.

As CD44 positively regulates the proliferation of breast cancer cells [31,41], we investigated whether combining the two drugs in concentrations affecting CD44 levels would have an impact on MDA-MB-231 cell propagation. This study found that a dose of 10 μg/mL fumiquinazoline F for 24 h did not significantly affect the cell number or CD44 levels. However, increasing the concentration of fumiquinazoline F resulted in significant changes in both cell growth and CD44 amount (Figure 8). The combined usage of low doses of fumiquinazoline F greatly enhanced the MG132’s inhibitory effect on cell growth, accompanied by a CD44 decrease. A combined treatment of MG132 with fumiquinazoline F at a concentration of 20 μg/mL results in a considerable reduction of live cells, indicating an additive action of the two compounds.

In addition to regulating cell proliferation, CD44 promotes tumor cell survival following pharmacological therapy [42,43,44]. As a result, we can assume that fumiquinazoline F and MG132 therapy inhibit MDA-MB-231 cell growth to some extent due to a decrease in CD44 levels. To summarize, our findings indicate that fumiquinazoline F can be employed as part of a combined treatment to inhibit breast tumor formation induced by triple-negative cancer cells.

## 3. Discussion

Breast cancer is a diverse group of tumors with varying etiologies, morphological characteristics, clinical outcomes, and therapeutic sensitivity. The anticancer characteristics of fumiquinazolines F and G were determined in this study using three breast cancer cell lines representing tumor cell types with varying levels of aggressiveness. As the major qualities of malignant cells are their ability to divide and migrate indefinitely, fumiquinazolines F and G were first tested for their capacity to prevent cell growth. Our cell growth suppression experiments yielded IC_50_ values of less than 100 μM. The hormone-dependent MCF-7 cell line responded equally to fumiquinazolines F and G, whereas the inhibitory activity of fumiquinazoline G was less prominent in the hormone-resistant BT474 and MDA-MB-231 cells (Table 1), indicating the difference between stereoisomers’ activity against various subtypes of breast cancer. In contrast to the hormone-insensitive prostate cell line PC3, hormone therapy-resistant breast cancer cells are able to respond to fumiquinazoline F-induced growth inhibition. Surprisingly, highly aggressive triple-negative MDA-MB-231 cells with multidrug resistance [45,46,47,48] show a sensitivity to fumiquinazoline F therapy with roughly the same efficacy as the least aggressive, tamoxifen-sensitive MCF-7 cells.

To investigate the processes involved in the growth inhibition of breast cancer cells treated with fumiquinazoline F, cell cycle distribution and cell death were examined. Our data show that the reduction of cell proliferation is more closely related to the retention of MDA-MB-231 cells in the G1 phase of the cell cycle than to apoptosis. These findings are consistent with prior research demonstrating that fumiquinazoline derivatives, such as 04NB-03 [1] and 4t-CHQB [13], cause cell cycle arrest in macrophage-like KG-1 cells in the G0/G1 phase. Considering the results obtained and the criterion that substances with an IC_50_ value of <100 μM are regarded as potential antitumor agents [49], we can conclude that fumiquinazoline F, which exhibits cytostatic properties alongside weak cytotoxicity, may be a viable candidate for the treatment of breast cancer characterized by various subtypes, including triple-negative MDA-MB-231-like cells.

Besides the unlimited proliferation, the second feature of tumor cells is their ability to migrate; therefore, the effect of fumiquinazoline F on the migration of MDA-MB-231 cells was investigated, and an IC_50_ value of 1.75 μg/mL (4.54 μM) was determined. Despite the lack of numerous published data about the range of IC_50_, which can be counted as potentially effective inhibitors of the process, it is known that IC_50_ values less than 10 μM are considered acceptable values [50,51]. Therefore, with regard to the inhibition of cell migration, fumiquinazoline F may be taken into account as a potential antitumor drug preventing breast cancer metastasis.

The increased ability of cancer cells to migrate and metastasize to distinct organs is closely related to their epithelial–mesenchymal transition [52,53,54]. EMT is a process characterized by a change in the phenotype of epithelial to mesenchymal cells. Detection of cells that have undergone EMT in tumor tissues is typical of the late stages of cancer development and is associated with an unfavorable prognosis for treatment and patient survival [55,56,57]. Furthermore, cancer mesenchymal cells have an increased ability to detach from surrounding cells, damage the basement membrane, travel via blood vessels, and frequently exhibit resistance to apoptosis and chemotherapy [55,58,59]. These functional changes are based on an alteration in the expression of a number of proteins: a decrease in the level of epithelial cadherin (E-cadherin) and an increase in that of vimentin, N-cadherin, fibronectin, and CD44, leading to a restructuring of the cytoskeleton and disruption of cell–cell contacts.

The current analysis indicated a reduction in vimentin and CD44 levels, accompanied by an increase in E-cadherin in MDA-MB-231 cells treated with fumiquinazoline F, but no alterations in their transcripts were observed. These findings raise the question of whether changes induced by fumiquinazoline F in EMT-related markers are independent or represent an interplay of these proteins in the control of their expression. CD44 has been shown to physically bind to the vimentin N-terminal head domain in HUVEC cells, and both proteins are overexpressed in cancer cells [60,61]. However, the functional effect of their interaction remains unknown. Previous research has demonstrated that, in esophageal carcinoma, the loss of E-cadherin is linked to the upregulation of CD44 [62]. On the other hand, E-cadherin participates in the negative regulation of CD44 expression and functional activity, because the increasing E-cadherin level in PC3 prostate cancer cells leads to a decrease in the amount of CD44 protein, and murine mammary carcinoma TA3 cells overexpressing E-cadherin display weakness in CD44-dependent binding with hyaluronic acid accompanied by a reduction in cell invasiveness [63,64]. Therefore, we can predict that, in MDA-MB-231 cells treated with fumiquinazolines F, a decrease or an increase in the level of one protein will have the opposite effect on the expression of another protein. However, it is still unclear whether CD44 or E-cadherin is the primary target for fumiquinazoline activity. It should be mentioned that E-cadherin is rapidly endocytosed and degraded via lysosomal and proteasomal pathways when it is not in complex with a p120 catenin. Furthermore, interaction of p120 with E-cadherin increases its half-life and protein abundance [65,66]. Hence, future research should determine if fumiquinazolines can enhance p120 catenin expression, leading to increased E-cadherin and decreased CD44 levels.

The proteasomal degradation pathway, whose activity can be inhibited by the proteasome inhibitor MG132, modulates the stability of vimentin and partially of E-cadherin [67,68,69], while degradation via lysosomes is considered the primary mechanism governing CD44 stability. However, the evidence pointing to CD44 accumulation in bladder and hepatic carcinoma cells following proteasome inhibitor MG132 treatment indicates a potential role for proteasomes in CD44 protein degradation [39,40]. In the current investigation, we expected to detect either no change or an increase in CD44 abundance in MDA-MB-231 cells incubated in the presence of MG132. However, the CD44 level decreased by roughly twofold in cells treated with 100 nM MG132 for 24 h (Figure 8). This result can be explained by changes in the levels of other proteins that control CD44 stability. For example, MARCH8, a member of the membrane-associated MARCH E3 ligase family, is implicated in the downregulation of CD44 via interaction and degradation of the complexes by the lysosomal degradation pathway [70,71]. The turnover of MARCH E3 ligase proteins is regulated through auto-ubiquitination and proteasomal activity, indicating that the inhibition of proteasome function could impede their degradation [70,72]. Consequently, we can assume that in MG132-treated MDA-MB-231 cells, elevated MARCH8 causes a reduction in the CD44 level.

Our data indicate that the proteasome inhibitor MG132, when applied in nanomolar quantities, effectively prevents MDA-MB-231 cell growth (Figure S4). This result is in agreement with previously published observations of high sensitivity to proteasome inhibition in basal-like TNBC cell lines such as MDA-MB-231 compared to luminal breast cancer subtypes like MCF-7 [73,74]. Furthermore, we discovered that low dosages of MG132 plus fumiquinazoline F considerably reduce cell viability compared to single drug application, implying that fumiquinazoline F can enhance MG132’s inhibitory effects (Figure 8C). This discovery raises the idea of testing fumiquinazolines in combination with clinically relevant proteasome inhibitors to improve TNBC therapy and overcome single-drug resistance. In addition, we found that fumiquinazoline F at doses over 10 μg/mL and MG132 at concentrations above 50 nM dramatically reduced CD44 protein levels in MDA-MB-231 cells. Notably, whereas when in lesser amounts a single compound did not impair the CD44 expression, a combination of both substances caused a decrease in the CD44 level (Figure 6 and Figure 8A,B). This result is noteworthy since CD44 is a well-known surface biomarker for cancer stem cells (CSCs) in a variety of tissues, including breast cancer [37,75,76]. CD44 regulates the primary functions of CSCs, including cell proliferation, migration, and invasion, as well as the ability to change the extracellular matrix of tissues to encourage new tumor formation. Cells expressing CD44 produce a higher amount of the cytokine transforming growth factor beta (TGFβ), which has been shown to initiate EMT [77,78]. Binding CD44 with its ligand HA triggers a signaling cascade including the stem cell maintenance transcription factor, Nanog, and ATP-binding cassette B1 (ABCB1), a drug efflux pump, contributing to multi-drug resistance [79,80,81]. The knockdown of CD44 reduces the CSC properties, alters the cell cycle and expression profiles of some stem cell-related genes, and induces differentiation of breast CSCs into cells with lower tumorigenic potential, which leads to an increase in their susceptibility to chemotherapy [82,83].

To date, various plant-derived alkaloids, such as berberine, matrine, dihydrocapsaicin, and piperine, have been identified as drugs having anti-metastasis properties in vitro and in vivo by blocking EMT and targeting CSCs; for review, see [84,85]. Fungi’s indole diterpene alkaloids derived from *Penicillium* species demonstrated good antiproliferation, antimigration, and anti-invasion capabilities against MDA-MB-231 and MCF-7 cells; nevertheless, their role in the regulation of EMT and breast cancer cell stemness remains unknown [86]. In the current research, we are the first to show that quinoline alkaloids fumiquinazolines F and G produced by the fungus *P. thymicola* possess anti-cancer properties against TNBC cells by modulating protein levels of EMT-related markers such as E-cadherin, vimentin, and CD44, causing MDA-MB-231 mesenchymal cells to revert to the epithelial phenotype seen in less malignant neoplasms. The fact that fumiquinazoline F did not impact the transcription of these genes laid the basis for further study of the putative mechanism of its action in the regulation of protein stability and degradation. Despite there being a long way to go before converting these compounds into clinical anti-cancer drugs, fumiquinazolines F and G can be employed as probes to investigate novel EMT-regulating pathways.

## 4. Materials and Methods

### 4.1. Cell Culture

Human breast cancer MCF-7, BT-474, and MDA-MB-231 cells, and prostate LNCap and PC3 cells, were obtained from the American Type Culture Collection (ATCC) (Manassas, VA, USA). DMEM, RPMI 1640, penicillin, streptomycin, and L-glutamine were bought from PanEco Ltd. (Moscow, Russia). Fetal bovine serum was from Biosera. Human breast cancer cells MCF-7, BT-474, and the triple negative cell line MDA-MB-231 were grown in DMEM. Prostate cancer cells LNCaP and PC-3 were cultivated in RPMI 1640. Both growth media were supplemented with 5% fetal bovine serum, 100 U/mL penicillin, 50 μg/mL streptomycin, and 2 mM L-glutamine. Cells were cultivated at 37 °C in a 5% CO_2_-humidified incubator.

### 4.2. Primers, Antibodies, and Reagents

The primer set shown below was used in RT-qPCR to analyze mRNA expression in MCF-7 and MDA-MB-231 cells. E-cadherin (CDH1, NM_004360): Forward-CCCAATACATCTCCCTTCACAG, Reverse-CCACCTCTAAGGCCATCTTTG; vimentin (VIM, NM_003380): Forward-CGTGAATACCAAGACCTGCTC, Reverse-GGAAAAGTTTGGAAGAGGCAG; SNAI2 (NM_003068): Forward-AGCATTTCAACGCCTCCA, Reverse-GGATCTCTGGTTGTGGTATGAC; ZEB2 (NM_014795): Forward-GCCATCTGATCCGCTCTTATC, Reverse-ACCTGTGTCCACTACATTGTC; GAPDH (NM_002046.7): Forward-ACATCGCTCAGACACCATG, Reverse-TGTAGTTGAGGTCAATGAAGGG; β-actin (ACTB, NM_001101): Forward-ACCTTCTACAATGAGCTGCG, Reverse-CCTGGATAGCAACGTACATGG.

The rabbit monoclonal antibodies anti-CD44 (Cell Signaling Technology, Danvers, MA, USA) and anti-β-actin, conjugated with peroxidase (Sigma, St. Louis, MO, USA), were used as primary antibodies. The peroxidase-conjugated antibodies against rabbit immunoglobulins (H&L, goat, Rockland, Limerick, PA, USA) were used as secondary antibodies for CD44 visualization in Western blot analysis. HRP-linked Super Signal West Pico chemiluminescent substrate was purchased from Thermo Fisher Scientific (Waltham, MA, USA). MG-132 (CAS 133407-82-6), cycloxecimide (CAS 66-81-9), and hyaluronic acid (CAS 9004-61-9) were purchased from Sigma, USA.

### 4.3. Fungal Strain Cultivation and Isolation of Fumiquinazolines F and G

The fungal strain *P. thymicola* VKM F-4453 (=FW-869) was obtained from the All-Russian Collection of Microorganisms (VKM), G.K. Skryabin Institute of Biochemistry and Physiology of Microorganisms, Russian Academy of Sciences. The metabolites fumiquinazolines F and G were extracted and purified from the culture fluid filtrate as described previously [12,87]. Briefly, the metabolites were recovered from the culture liquid filtrate by three-fold extraction with chloroform at pH 5, and a total of 390 mg of extract was obtained. The metabolites were isolated by column chromatography on silica gel (Silica gel 60, 0.063–0.1 mm, Merck, Darmstadt, Germany). Elution was performed with a gradient of CHCl_3_ and CHCl_3_:MeOH (9:1) to obtain six fractions. Fraction 2 (75 mg) contained fumiquinazoline F, and fraction 3 (32 mg) included a mixture of fumiquinazolines F and G. Fumiquinazolines in fraction 3 were separated by preparative thin-layer chromatography (TLC) using 20 × 20 cm silica gel plates (Silica gel F254, Merck, Darmstadt, Germany) in solvent systems CHCl_3_:MeOH (9:1). Finally, 12 mg of fumiquinazoline F and 17 mg of fumiquinazoline G were recovered from fraction 3. A total of 87 mg of fumiquinazoline F and 17 mg of fumiquinazoline G were isolated from 390 mg of culture liquid extract. TLC, UV, and MS assays revealed a purity level of >98%.

### 4.4. Cell Growth Assay

The experiments were performed using the xCELLigence DP system (ACEA Biosciences, San Diego, CA, USA) as described previously [88]. In brief, 100 μL of cells (10,000 cells/well) in the growth medium was seeded in triplicate in E-plates. The plates were placed in the xCELLigence instrument to continuously record the impedance on the microelectrodes. After 24 h, 100 μL of DMEM (for MCF-7, BT474, and MDA-MB-231) or RPMI 1640 (for LNCap and PC-3) growth medium containing either DMSO (vehicle control) or fumiquinazolines F and G at the indicated concentrations was added to the wells. Changes in electrical impedance presented as a cell index (CI) were measured every 15 min. IC_50_ values were calculated by xCELLigence RTCA Software Pro, which uses the CI values detected at different (0–30 μg/mL) ranges of drug concentration after 24, 48, or 72 h of compound application.

A cell growth assay with crystal violet staining as described [89] was used to measure the inhibitory effects of fumiquinazoline F and MG-132 on MDA-MB-231 cells. In brief, MDA-MB-231 at 1 × 10^4^ cells per well in 100 μL of the growth media was plated in quadruplicates in 96-well plates. The next day, 100 μL of the media containing fumiquinazoline F, MG132, or their combinations was applied to the cells to achieve the final concentrations indicated in Figure 8. After incubation for 24 h, the growth medium was discarded, and 100 μL of 0.1% crystal violet (Acros Organic, Geel, Belgium) in 25% methanol was added to each well for 20 min. Cells were washed three times with water and allowed to dry. The dye was then extracted from cells by 200 μL of 10% acetic acid, and the absorbance of this solution was measured at 590 nm using a Multi-Mode Microplate Reader FilterMax F5 and SoftMax Pro 7 software (Molecular Devices, San Jose, CA, USA).

### 4.5. Cell Death Assay and Cell Cycle Analysis

MDA-MB-231 cells were seeded at 3 × 10^5^ cells in six-well cell culture plates with 2 mL of 5% FBS DMEM. The next day, 2 mL of 5% FBS DMEM with or without fumiquinazoline F was added to a final concentration of 10, 20, and 30 μg/mL, and cells were incubated for 48 h. Following the incubation, floating and attached cells were collected and pooled by centrifugation at 290× *g* (Eppendorf, Hamburg, Germany, Centrifuge 5810 R) for 10 min. The collected cells were stained using the Annexin V-AF 488/PI Apoptotic Cell Detection Kit (Lumiprobe, Moscow, Russia) according to the manufacturer’s instructions (https://www.lumiprobe.com/manual/annexin-v-af-pi-apoptosis-kit (accessed on 22 January 2025)). Stained cells were examined using a Novocyte flow cytometer (Agilent, Santa Clara, CA, USA) and NovoExpress flow cytometry software (version 1.6.3) (Agilent, Santa Clara, CA, USA).

For cell cycle analysis, propidium iodide staining followed by flow cytofluorometric analysis was used [90]. MDA-MB-231 cells were plated at 8 × 10^5^ cells in 60 mm cell culture dishes containing 4 mL of 5% FBS DMEM. The next day, the cells were rinsed with phosphate-buffered saline (PBS) and incubated in the serum-free DMEM. Following 3 h of incubation, the serum-free medium was replaced with the growth media supplemented with DMSO (vehicle control) or fumiquinazoline F at the doses shown in Figure 2B. After 24 h of incubation, floating and attached cells were collected, centrifuged at 290× *g* for 10 min, and washed with PBS. The pelleted cells were resuspended in 1 mL of cold 70% ethanol and stored at −20 °C for 24 h. The fixed cells were centrifuged at 453× *g* for 15 min, rehydrated with PBS, and resuspended in 1 mL of PBS buffer containing 100 μg/mL RNase and 25 μg/mL propidium iodide. Following a 30 min incubation at 37 °C, the cell suspensions were examined using a Novocyte flow cytometer (Agilent, USA) and Agilent NovoExpress cell cycle software.

### 4.6. Cell Migration Assay

The cell migration rate was monitored in real time using the xCELLigence system (CIM plates) [88]. MDA-MB-231 (30,000 cells) were seeded in serum-free DMEM containing DMSO or fumiquinazoline F at the indicated concentrations in each well of the upper chamber of the CIM plate. The DMEM medium supplemented with 5% FBS was added to each well of the lower chamber. The serum served as a chemoattractant, which stimulated cell migration through the porous membrane. The impedance of each well was monitored by the xCELLigence system every 10 min for 25 h and displayed as CI values reflecting the number of cells that migrated from the upper chamber through the membrane to the lower chamber toward the medium containing the chemoattractant.

For the wound-healing assay [91], the monolayer of MDA-MB-231 cells in 30 mm cell culture dishes was damaged by scraping the cells along the line with a pipette tip. After wounding, the monolayer was washed twice with PBS, and 2 mL of DMEM containing 1.5% FBS with or without fumiquinazoline F at the indicated concentration was added to the plates. Then, a micrograph of the cell-free region was taken using a MICROMED E-LUM microscope (Micromed, Moscow, Russia). The same region was photographed after 24 h of incubation. Cell migration was estimated by measuring the distance between two layers of migrated cells in the gap closure region using ImageJ software version 1.53 (NIH, Bethesda, MD, USA).

### 4.7. ELISA

MDA-MB-231 cells were seeded in the growth media at 4 × 10^6^ cells per 10 cm per tissue culture plate, and fumiquinazolines were added the next day at final concentrations of 0, 1.75, 5, 10, and 15 μg/mL. The cells were cultured for either 48 or 300 h at 37 °C, 5% CO_2_ in a humidified atmosphere. For long-time incubation, cells were re-plated every 72 h (dilution 1:3) in a fresh medium with fumiquinazolines or DMSO. At the end of the treatment, cells were collected and lysed using the lysis buffer supplied by the manufacturer. Vimentin (VIM, SEB040Hu) and E-cadherin (SEA017Hu) levels were quantified in cell lysates utilizing ELISA kits (Cloud-Clone Corp., Houston, TX, USA) in accordance with the manufacturer’s procedure. The absorbance was measured at 450 nm using a Multi-Mode Microplate Reader FilterMax F5 and SoftMax Pro 7 software for Windows 10 and 11 (Molecular Devices, San Jose, CA, USA).

### 4.8. RT-qPCR

MDA-MB-231 cells (4 × 10^6^) were plated and treated for 48 h with either fumiquinazoline F or DMSO (vehicle control) as described above. After incubation, the growth medium was removed and the cells were washed with chilled PBS and lysed in 1 mL of TRIzol Reagent (Invitrogen, Carlsbad, CA, USA). RNA was then extracted according to the manufacturer’s protocol. The concentration of RNA was determined using a NanoPhotometer P-Class P360 (Implen, München, Germany). The iScript Reverse Transcription Supermix for RT-qPCR kit (Bio-Rad, Hercules, CA, USA) was used for first-strand cDNA synthesis. The reagent kit for RT-PCR in the presence of SYBR Green (Syntol, Moscow, Russia) and a DTlite detection amplifier (DNA-Technology, Moscow, Russia) with built-in software was applied for gene expression analysis in accordance with the manufacturer’s instructions. Amplified cDNA of GAPDH and β-actin was utilized for normalization of the relative gene expression using the 2^−ΔΔCt^ method [92].

### 4.9. Western Blot Analysis

MDA-MB-231 cells were seeded at 4 × 10^5^ cells/well in six-well cell culture plates in a DMEM growth medium. The next day, the medium was replaced with DMEM containing either fumiquinazoline F at various concentrations or DMSO as a vehicle control, and cultivation continued for 48 h or 24 h when the incubation with fumiquinazoline F and MG132 was used. At the end of the treatment, cells were washed with cold PBS, lysed in 200 μL RIPA lysis buffer supplemented with 1 mM phenylmethylsulfonyl fluoride and protease inhibitor cocktail Set V (Calbiochem, San Diego, CA, USA), and then the protein concentration was measured using the BCA™ Protein Assay Kit (Thermo Fisher Scientific, Waltham, MA, USA). Up to 25 μg of total protein per sample was separated using 10% 1.5 mm SDS-PAGE and transferred to a nitrocellulose membrane (Bio-Rad Laboratories, Hercules, CA, USA). The membrane was blocked with 5% skim milk in TBST (20 mM Tris-HCl, pH 7.5, 150 mM NaCl, 0.1% Tween 20). Then, it was incubated overnight at 4 °C with anti-CD44 antibodies (1:1000 dilution in TBST with 5% bovine serum albumin). After three washes in TBST, the membrane was incubated for 1 h in 5% skim milk/TBST containing peroxidase-linked secondary antibodies (1:5000 dilution). The membrane was then washed three times with TBST. Protein detection was carried out according to the recommended protocol for the SuperSignal West Pico chemiluminescent substrate (Thermo Scientific, Waltham, MA, USA). At the end of the procedure, the membrane was washed three times in TBST, re-incubated with anti-β-actin HRP-conjugated antibodies (1:5000 dilution) for 1 h at room temperature, and protein identification was performed as previously described. The relative protein expression levels were evaluated by densitometry of Western blot images using Image studio software version 5 (LI-COR Biosciences, Lincoln, NE, USA).

### 4.10. Cycloheximide Chase Assay

MDA-MB-231 cells were plated at 4 × 10^5^ cells per well in eight wells across two six-well cell culture plates and incubated in growth conditions for 24 h. On the following day, the media in four wells was substituted with DMEM containing DMSO (vehicle control), while fumiquinazoline F (10 μg/mL) was introduced into four additional wells. The cells were subsequently grown for 24 h at 37 °C and 5% CO_2_ in a humidified environment. Then, every eight hours, cycloheximide was added to a final concentration of 50 μg/mL into a pair of wells containing either DMSO or fumiquinazoline F, and incubation was continued for 24 h. Thus, the overall treatment time with fumiquinazoline F was 48 h, whereas the presence of cycloheximide was varied from 8 and 16 to 24 h. At the end of the incubation, all cells were lysed, and the CD44 level was determined by immunoblotting as stated in the Section 4.9.

### 4.11. Hyaluronic Acid Adhesion Assay

The cell adhesion assay was carried out as previously described [93]. In brief, prior to the experiment, wells of a 96-well plate were coated with HA by adding 100 μL per well of hyaluronic acid solution (1 mg/mL) in PBS, followed by 24 h incubation at + 4 °C. Then, wells were rinsed with PBS and air-dried. MDA-MB-231 cells (4 × 10^5^ cells/well) were pretreated with fumiquinazoline F for 48 h as stated in the Section 4.9. At the end of the treatment, cells were harvested, suspended in serum-free DMEM, and plated in quadruplicates at 5 × 10^4^ cells/well into HA-pre-coated wells and left to adhere for 30 min at 37 °C and 5% CO_2_ in a humidified atmosphere. The wells were then washed twice with PBS, and attached cells were subsequently fixed with 4% paraformaldehyde solution in PBS for 20 min. The quantitation of attached cells by staining with 0.1% crystal violet was carried out as stated in the Section 4.4.

### 4.12. Statistical Analysis

All experiments were conducted at least twice, employing several purifications of fumiquinazolines to validate cellular responses. The results of quantitative studies are reported as the means ± SD. Differences were analyzed by the Student’s *t*-test, and *p* values of < 0.05 were considered significant.

## Figures and Tables

**Table 1:** IC_50_ values for breast and prostate cancer cells treated with fumiquinazoline F (F) or fumiquinazoline G (G) after 24–72 h of incubation. Data were obtained using xCELLigence RTCA Software Pro.

Cell Lines and Compounds	IC_50_ Values—24 h	IC_50_ Values—48 h	IC_50_ Values—72 h
MCF-7 F	21.2 μg/mL (59.1 μM)	17.2 μg/mL (48 μM)	18 μg/mL (50.3 μM)
MCF-7 G	20.3 μg/mL (56.7 μM)	14.5 μg/mL (40.4 μM)	15.6 μg/mL (43.5 μM)
BT-474 F	23.4 μg/mL (65.2 μM)	19.9 μg/mL (55.5 μM)	16.5 μg/mL (46 μM)
BT-474 G	>100 μM	>100 μM	30.3 μg/mL (84.4 μM)
MDA-MB-231 F	21.5 μg/mL (60.1 μM)	19.4 μg/mL (54.1 μM)	23.5 μg/mL (65.5 μM)
MDA-MB-231 G	34.2 μg/mL (95.5 μM)	31.6 μg/mL (88.2 μM)	>100 μM
LNCaP F	21.6 μg/mL (60.2 μM)	17 μg/mL (47.4 μM)	22.8 μg/mL (63.7 μM)
LNCaP G	29.2 μg/mL (81.5 μM)	17.1 μg/mL (47.6 μM)	21.4 μg/mL (59.8 μM)
PC-3 F	>100 μM	>100 μM	>100 μM
PC-3 G	>100 μM	>100 μM	>100 μM

## Data Availability

The raw data supporting the conclusions of this article will be made available by the authors on request.

## Supplementary Materials

The following supporting information can be downloaded at: https://www.mdpi.com/article/10.3390/ijms26157582/s1.

## References

[1] Wang R., Zhu Y., Chen J., Wang Y., Song X., Wu Y., Jin F., Wang Y. (2021). The Quinazoline Derivative, 04NB-03, Induces Cell Cycle Arrest and Apoptosis in Hepatocellular Carcinoma Cells in a Reactive Oxygen Species-Dependent Manner. Chem. Biol. Interact..

[2] Sharifi-Rad J., Ozleyen A., Boyunegmez Tumer T., Oluwaseun Adetunji C., El Omari N., Balahbib A., Taheri Y., Bouyahya A., Martorell M., Martins N. (2019). Natural Products and Synthetic Analogs as a Source of Antitumor Drugs. Biomolecules.

[3] Boulis A.G., Hamed A.A., El-Awady M.E., Mohamed A.R., Eliwa E.M., Asker M.M.S., Shaaban M. (2020). Diverse Bioactive Metabolites from *Penicillium* sp. MMA Derived from the Red Sea: Structure Identification and Biological Activity Studies. Arch. Microbiol..

[4] Zhang X., Yin Q., Li X., Liu X., Lei H., Wu B. (2022). Structures and Bioactivities of Secondary Metabolites from Penicillium Genus Since 2010. Fitoterapia.

[5] Shang X.F., Morris-Natschke S.L., Liu Y.Q., Guo X., Xu X.-S., Goto M., Li J.-C., Yang G.-Z., Lee K.-H. (2018). Biologically Active Quinoline and Quinazoline Alkaloids Part I. Med. Res. Rev..

[6] Kaur T., Bhandari D.D. (2023). Annotated Review on Various Biological Activities of Quinoline Molecule. Biointerface Res. Appl. Chem..

[7] Dai L.H., Zhang G.R., Ou Y.H., Liu X.J., Yao H.L., Hu W.H., Li H.J., Lan W.J. (2025). Five New Indole Alkaloid Derivatives from Deep-Sea Fungus Aspergillus fumigatus AF1. Mar. Drugs.

[8] Almeida M.C., Szemerédi N., Durães F., Long S., Resende D.I.S.P., Martins da Costa P., Pinto M., Spengler G., Sousa E. (2023). Effect of Indole-Containing Pyrazino [2,1-b]quinazoline-3,6-diones in the Virulence of Resistant Bacteria. Antibiotics.

[9] Resende D.I.S.P., Boonpothong P., Sousa E., Kijjoa A., Pinto M.M.M. (2019). Chemistry of the Fumiquinazolines and Structurally Related Alkaloids. Nat. Prod. Rep..

[10] Han X., Xu X.Y., Cui C.B., Gu Q. (2007). Alkaloidal Compounds Produced by a Marine-Derived Fungus, Aspergillus fumigatus H1-04, and Their Antitumor Activities. Chin. J. Med. Chem..

[11] Zhou Y.M., Debbab A., Mandi A., Wray V., Schulz B., Muller W.E.G., Kassack M., Lin W.H., Kurtan T., Proksch P. (2013). Alkaloids from the Sponge-Associated Fungus *Aspergillus* sp.. Appl. Environ. Microbiol..

[12] Zhelifonova V.P., Antipova T.V., Kozlovskii A.G. (2012). Biosynthesis of Fumiquinazolines by the Fungus *Penicillium thymicola*. Appl. Biochem. Microbiol..

[13] Rahimian A., Mahdavi M., Rahbarghazi R., Charoudeh H.N. (2019). 4t-CHQ, a Spiro-Quinazolinone Benzenesulfonamide Derivative, Induces G0/G1 Cell Cycle Arrest and Triggers Apoptosis Through Down-Regulation of Survivin and Bcl2 in the Leukemia Stem-Like KG1-a Cells. Anticancer Agents Med. Chem..

[14] Shang X.F., Morris-Natschke S.L., Liu Y.Q., Li X.H., Zhang J.Y., Lee K.H. (2022). Biology of Quinoline and Quinazoline Alkaloids. Alkaloids Chem. Biol..

[15] Lee A.V., Oesterreich S., Davidson N.E. (2015). MCF-7 Cells—Changing the Course of Breast Cancer Research and Care for 45 Years. J. Natl. Cancer Inst..

[16] Dai X., Cheng H., Bai Z., Li J. (2017). Breast Cancer Cell Line Classification and Its Relevance with Breast Tumor Subtyping. J. Cancer.

[17] Zuo T., Wang L., Morrison C., Chang X., Zhang H., Li W., Liu Y., Wang Y., Liu X., Chan M.W. (2007). FOXP3 Is an X-Linked Breast Cancer Suppressor Gene and an Important Repressor of the HER-2/ErbB2 Oncogene. Cell.

[18] Witt B.L., Tollefsbol T.O. (2023). Molecular, Cellular, and Technical Aspects of Breast Cancer Cell Lines as a Foundational Tool in Cancer Research. Life.

[19] Nadanaka S., Tamura J.I., Kitagawa H. (2022). Chondroitin Sulfates Control Invasiveness of the Basal-Like Breast Cancer Cell Line MDA-MB-231 Through ROR1. Front. Oncol..

[20] Yin L., Duan J.J., Bian X.W., Yu S.C. (2020). Triple-Negative Breast Cancer Molecular Subtyping and Treatment Progress. Breast Cancer Res..

[21] Rubenstein M., Hollowell C.M., Guinan P. (2011). In LNCaP Cells Enhanced Expression of Both Androgen Receptor and Costimulatory Protein p300 Compensate for Antisense Oligonucleotide Suppression of bcl-2. Ther. Adv. Urol..

[22] Abate-Shen C., Nunes de Almeida F. (2022). Establishment of the LNCaP Cell Line—The Dawn of an Era for Prostate Cancer Research. Cancer Res..

[23] Tai S., Sun Y., Squires J.M., Zhang H., Oh W.K., Liang C.Z., Huang J. (2011). PC3 Is a Cell Line Characteristic of Prostatic Small Cell Carcinoma. Prostate.

[24] Niu Y., Yeh S., Miyamoto H., Li G., Altuwaijri S., Yuan J., Han R., Ma T., Kuo H.C., Chang C. (2008). Tissue Prostate-Specific Antigen Facilitates Refractory Prostate Tumor Progression via Enhancing ARA70-Regulated Androgen Receptor Transactivation. Cancer Res..

[25] Mielczarek L., Krug P., Mazur M., Milczarek M., Chilmonczyk Z., Wiktorska K. (2019). In the Triple-Negative Breast Cancer MDA-MB-231 Cell Line, Sulforaphane Enhances the Intracellular Accumulation and Anticancer Action of Doxorubicin Encapsulated in Liposomes. Int. J. Pharm..

[26] Lucantoni F., Lindner A.U., O’Donovan N., Prehn J.H.M. (2018). Systems Modeling Accurately Predicts Responses to Genotoxic Agents and Their Synergism with BCL-2 Inhibitors in Triple Negative Breast Cancer Cells. Cell Death Dis..

[27] Huang Z., Yu P., Tang J. (2020). Characterization of Triple-Negative Breast Cancer MDA-MB-231 Cell Spheroid Model. Onco Targets Ther..

[28] Isert L., Mehta A., Loiudice G., Oliva A., Roidl A., Merkel O.M. (2023). An In Vitro Approach to Model EMT in Breast Cancer. Int. J. Mol. Sci..

[29] Wei Z., Shan Z., Shaikh Z.A. (2018). Epithelial-Mesenchymal Transition in Breast Epithelial Cells Treated with Cadmium and the Role of Snail. Toxicol. Appl. Pharmacol..

[30] Hu S., Shi X., Liu Y., He Y., Du Y., Zhang G., Yang C., Gao F. (2020). CD44 Cross-Linking Increases Malignancy of Breast Cancer via Upregulation of p-Moesin. Cancer Cell Int..

[31] Chen C., Zhao S., Karnad A., Freeman J.W. (2018). The Biology and Role of CD44 in Cancer Progression: Therapeutic Implications. J. Hematol. Oncol..

[32] Al-Othman N., Alhendi A., Ihbaisha M., Barahmeh M., Alqaraleh M., Al-Momany B.Z. (2019). Role of CD44 in Breast Cancer. Breast Dis..

[33] Olsson E., Honeth G., Bendahl P.O., Saal L.H., Gruvberger-Saal S., Ringnér M., Vallon-Christersson J., Jönsson G., Holm K., Lövgren K. (2011). CD44 Isoforms Are Heterogeneously Expressed in Breast Cancer and Correlate with Tumor Subtypes and Cancer Stem Cell Markers. BMC Cancer.

[34] Ryoo I.G., Choi B.H., Ku S.K., Kwak M.K. (2018). High CD44 Expression Mediates p62-Associated NFE2L2/NRF2 Activation in Breast Cancer Stem Cell-Like Cells: Implications for Cancer Stem Cell Resistance. Redox Biol..

[35] Birzele F., Voss E., Nopora A., Honold K., Heil F., Lohmann S., Verheul H., Le Tourneau C., Delord J.P., van Herpen C. (2015). CD44 Isoform Status Predicts Response to Treatment with Anti-CD44 Antibody in Cancer Patients. Clin. Cancer Res..

[36] Luo Y., Tian Z., Hua X., Huang M., Xu J., Li J., Huang H., Cohen M., Huang C. (2020). Isorhapontigenin (ISO) Inhibits Stem Cell-Like Properties and Invasion of Bladder Cancer Cell by Attenuating CD44 Expression. Cell. Mol. Life Sci..

[37] McFarlane S., Coulter J.A., Tibbits P., O’Grady A., McFarlane C., Montgomery N., Hill A., McCarthy H.O., Young L.S., Kay E.W. (2015). CD44 Increases the Efficiency of Distant Metastasis of Breast Cancer. Oncotarget.

[38] Chang G., Wang J., Zhang H., Zhang Y., Wang C., Xu H., Zhang H., Lin Y., Ma L., Li Q. (2014). CD44 Targets Na(+)/H(+) Exchanger 1 to Mediate MDA-MB-231 Cells’ Metastasis via the Regulation of ERK1/2. Br. J. Cancer.

[39] Liu W., Ji Z., Wu B., Huang S., Chen Q., Chen X., Wei Y., Jiang J. (2021). Siglec-15 Promotes the Migration of Liver Cancer Cells by Repressing Lysosomal Degradation of CD44. FEBS Lett..

[40] Haakenson J.K., Khokhlatchev A.V., Choi Y.J., Linton S.S., Zhang P., Zaki P.M., Fu C., Cooper T.K., Manni A., Zhu J. (2015). Lysosomal Degradation of CD44 Mediates Ceramide Nanoliposome-Induced Anoikis and Diminished Extravasation in Metastatic Carcinoma Cells. J. Biol. Chem..

[41] Nam K., Oh S., Lee K.M., Yoo S.A., Shin I. (2015). CD44 Regulates Cell Proliferation, Migration, and Invasion via Modulation of c-Src Transcription in Human Breast Cancer Cells. Cell Signal..

[42] Ravindranath A.K., Kaur S., Wernyj R.P., Kumaran M.N., Miletti-Gonzalez K.E., Chan R., Lim E., Madura K., Rodriguez-Rodriguez L. (2015). CD44 Promotes Multi-Drug Resistance by Protecting P-Glycoprotein from FBXO21-Mediated Ubiquitination. Oncotarget.

[43] Xu H., Niu M., Yuan X., Wu K., Liu A. (2020). CD44 as a Tumor Biomarker and Therapeutic Target. Exp. Hematol. Oncol..

[44] Vargas C., Aguirre-Ducler A., Cereceda K., Quijada S., Escobar-Gómez N., Castillo R.L., Escobar-Aguirre M. (2025). CD44 Marks Dormant Tumor Cells After HER2 Inhibition in Breast Cancer Cells. Int. J. Mol. Sci..

[45] Chen J., Lu L., Feng Y., Wang H., Dai L., Yan li, Zhang P. (2011). PKD2 Mediates Multi-Drug Resistance in Breast Cancer Cells Through Modulation of P-Glycoprotein Expression. Cancer Lett..

[46] Błaszczak E., Miziak P., Odrzywolski A., Baran M., Gumbarewicz E., Stepulak A. (2025). Triple-Negative Breast Cancer Progression and Drug Resistance in the Context of Epithelial–Mesenchymal Transition. Cancers.

[47] Zwartsen A., Chottanapund S., Kittakoop P., Navasumrit P., Ruchirawat M., Van Duursen M.B.M., Van den Berg M. (2019). Evaluation of Anti-Tumour Properties of Two Depsidones—Unguinol and Aspergillusidone D—In Triple-Negative MDA-MB-231 Breast Tumour Cells. Toxicol. Rep..

[48] Simu S., Marcovici I., Dobrescu A., Malita D., Dehelean C.A., Coricovac D., Olaru F., Draghici G.A., Navolan D. (2021). Insights into the Behavior of Triple-Negative MDA-MB-231 Breast Carcinoma Cells Following the Treatment with 17β-Ethinylestradiol and Levonorgestrel. Molecules.

[49] Wong C.C., Cheng K.W., Rigas B. (2012). Preclinical Predictors of Anticancer Drug Efficacy: Critical Assessment with Emphasis on Whether Nanomolar Potency Should Be Required of Candidate Agents. J. Pharmacol. Exp. Ther..

[50] Hughes J.P., Rees S., Kalindjian S.B., Philpott K.L. (2011). Principles of Early Drug Discovery. Br. J. Pharmacol..

[51] Williams A., Hyland R., Jones B.C., Smith D.A., Hurst S., Goosen T.C., Peterkin V., Koup J.R., Ball S.E. (2004). Drug-Drug Interactions for UDP-Glucuronosyltransferase Substrates: A Pharmacokinetic Explanation for Typically Observed Low Exposure (AUCi/AUC) Ratios. Drug Metab. Dispos..

[52] Thiery J.P. (2002). Epithelial-Mesenchymal Transitions in Tumour Progression. Nat. Rev. Cancer.

[53] Liang L., Kaufmann A.M. (2023). The Significance of Cancer Stem Cells and Epithelial-Mesenchymal Transition in Metastasis and Anti-Cancer Therapy. Int. J. Mol. Sci..

[54] Liao T.T., Yang M.H. (2017). Revisiting Epithelial-Mesenchymal Transition in Cancer Metastasis: The Connection Between Epithelial Plasticity and Stemness. Mol. Oncol..

[55] Mittal V. (2018). Epithelial Mesenchymal Transition in Tumor Metastasis. Annu. Rev. Pathol..

[56] Huang Y., Hong W., Wei X. (2022). The Molecular Mechanisms and Therapeutic Strategies of EMT in Tumor Progression and Metastasis. J. Hematol. Oncol..

[57] Busch E.L., Don P.K., Chu H., Richardson D.B., Keku T.O., Eberhard D.A., Avery C.L., Sandler R.S. (2018). Diagnostic Accuracy and Prediction Increment of Markers of Epithelial-Mesenchymal Transition to Assess Cancer Cell Detachment from Primary Tumors. BMC Cancer.

[58] Pérez-González A., Bévant K., Blanpain C. (2023). Cancer Cell Plasticity During Tumor Progression, Metastasis and Response to Therapy. Nat. Cancer.

[59] Atiya H.I., Gorecki G., Garcia G.L., Frisbie L.G., Baruwal R., Coffman L. (2023). Stromal-Modulated Epithelial-to-Mesenchymal Transition in Cancer Cells. Biomolecules.

[60] Päll T., Pink A., Kasak L., Turkina M., Anderson W., Valkna A., Kogerman P. (2011). Soluble CD44 Interacts with Intermediate Filament Protein Vimentin on Endothelial Cell Surface. PLoS ONE.

[61] Steinmetz N.F., Maurer J., Sheng H., Bensussan A., Maricic I., Kumar V., Braciak T.A. (2011). Two Domains of Vimentin Are Expressed on the Surface of Lymph Node, Bone and Brain Metastatic Prostate Cancer Lines along with the Putative Stem Cell Marker Proteins CD44 and CD133. Cancers.

[62] Le Bras G.F., Allison G.L., Richards N.F., Ansari S.S., Washington M.K., Andl C.D. (2011). CD44 Upregulation in E-Cadherin-Negative Esophageal Cancers Results in Cell Invasion. PLoS ONE.

[63] Deep G., Jain A.K., Ramteke A., Ting H., Vijendra K.C., Gangar S.C., Agarwal C., Agarwal R. (2014). SNAI1 Is Critical for the Aggressiveness of Prostate Cancer Cells with Low E-Cadherin. Mol. Cancer.

[64] Xu Y., Yu Q. (2003). E-Cadherin Negatively Regulates CD44-Hyaluronan Interaction and CD44-Mediated Tumor Invasion and Branching Morphogenesis. J. Biol. Chem..

[65] Hatzfeld M. (2005). The p120 Family of Cell Adhesion Molecules. Eur. J. Cell Biol..

[66] Kourtidis A., Ngok S.P., Anastasiadis P.Z. (2013). p120 Catenin: An Essential Regulator of Cadherin Stability, Adhesion-Induced Signaling, and Cancer Progression. Prog. Mol. Biol. Transl. Sci..

[67] Park S.Y., Yoon S., Sun E.G., Zhou R., Bae J.A., Seo Y.W., Chae J.I., Paik M.J., Ha H.H., Kim H. (2017). Glycoprotein 90K Promotes E-Cadherin Degradation in a Cell Density-Dependent Manner via Dissociation of E-Cadherin-p120-Catenin Complex. Int. J. Mol. Sci..

[68] Korolchuk V.I., Mansilla A., Menzies F.M., Rubinsztein D.C. (2009). Autophagy Inhibition Compromises Degradation of Ubiquitin-Proteasome Pathway Substrates. Mol. Cell.

[69] Pang K., Park J., Ahn S.G., Lee J., Park Y., Ooshima A., Mizuno S., Yamashita S., Park K.-S., Lee S.-Y. (2019). RNF208, an Estrogen-Inducible E3 Ligase, Targets Soluble Vimentin to Suppress Metastasis in Triple-Negative Breast Cancers. Nat. Commun..

[70] Chen W., Patel D., Jia Y., Yu Z., Liu X., Shi H., Liu H. (2021). MARCH8 Suppresses Tumor Metastasis and Mediates Degradation of STAT3 and CD44 in Breast Cancer Cells. Cancers.

[71] Eyster C.A., Cole N.B., Petersen S., Viswanathan K., Früh K., Donaldson J.G. (2011). MARCH Ubiquitin Ligases Alter the Itinerary of Clathrin-Independent Cargo from Recycling to Degradation. Mol. Biol. Cell.

[72] Bourgeois-Daigneault M.C., Thibodeau J. (2012). Autoregulation of MARCH1 Expression by Dimerization and Autoubiquitination. J. Immunol..

[73] Larsson P., Pettersson D., Olsson M., Sarathchandra S., Abramsson A., Zetterberg H., Ittner E., Forssell-Aronsson E., Kovács A., Karlsson P. (2024). Repurposing Proteasome Inhibitors for Improved Treatment of Triple-Negative Breast Cancer. Cell Death Discov..

[74] Byers H.A., Brooks A.N., Vangala J.R., Grible J.M., Feygin A., Clevenger C.V., Harrell J.C., Radhakrishnan S.K. (2023). Evaluation of the NRF1-Proteasome Axis as a Therapeutic Target in Breast Cancer. Sci. Rep..

[75] Chang S.J., Ou-Yang F., Tu H.P., Lin C.H., Huang S.H., Kostoro J., Hou M.F., Chai C.Y., Kwan A.L. (2016). Decreased Expression of Autophagy Protein LC3 and Sternness (CD44+/CD24-/low) Indicate Poor Prognosis in Triple-Negative Breast Cancer. Hum. Pathol..

[76] Vadhan A., Hou M.F., Vijayaraghavan P., Wu Y.C., Hu S.C., Wang Y.M., Cheng T.L., Wang Y.Y., Yuan S.F. (2022). CD44 Promotes Breast Cancer Metastasis through AKT-Mediated Downregulation of Nuclear FOXA2. Biomedicines.

[77] Vira D., Basak S.K., Veena M.S., Batra R.K., Srivatsan E.S. (2012). Cancer Stem Cells, microRNAs, and Therapeutic Strategies Including Natural Products. Cancer Metastasis Rev..

[78] Nakano M., Ito M., Tanaka R., Ariyama H., Mitsugi K., Makiyama A., Uchino K., Esaki T., Tsuruta N., Hanamura F. (2018). Epithelial-Mesenchymal Transition Is Activated in CD44-Positive Malignant Ascites Tumor Cells of Gastrointestinal Cancer. Cancer Sci..

[79] Zöller M. (2011). CD44: Can a Cancer-Initiating Cell Profit from an Abundantly Expressed Molecule?. Nat. Rev. Cancer.

[80] Bourguignon L.Y., Peyrollier K., Xia W., Gilad E. (2008). Hyaluronan-CD44 Interaction Activates Stem Cell Marker Nanog, Stat-3-Mediated MDR1 Gene Expression, and Ankyrin-Regulated Multidrug Efflux in Breast and Ovarian Tumor Cells. J. Biol. Chem..

[81] Chanmee T., Ontong P., Kimata K., Itano N. (2015). Key Roles of Hyaluronan and Its CD44 Receptor in the Stemness and Survival of Cancer Stem Cells. Front. Oncol..

[82] Pham P.V., Phan N.L., Nguyen N.T., Truong N.H., Duong T.T., Le D.V., Truong K.D., Phan N.K. (2011). Differentiation of Breast Cancer Stem Cells by Knockdown of CD44: Promising Differentiation Therapy. J. Transl. Med..

[83] Chu X., Tian W., Ning J., Xiao G., Zhou Y., Wang Z., Zhai Z., Tanzhu G., Yang J., Zhou R. (2024). Cancer Stem Cells: Advances in Knowledge and Implications for Cancer Therapy. Signal Transduct. Target. Ther..

[84] Liu S., Li L., Ren D. (2023). Anti-Cancer Potential of Phytochemicals: The Regulation of the Epithelial-Mesenchymal Transition. Molecules.

[85] Taylor W.F., Jabbarzadeh E. (2017). The Use of Natural Products to Target Cancer Stem Cells. Am. J. Cancer Res..

[86] Yu J., Wang X., Du P., Shi H. (2024). The Therapeutic Potential and Application of Marine Alkaloids in Treating Breast Cancer. Front. Mar. Sci..

[87] Kozlovskii A.G., Zhelifonova V.P., Antipova T.V., Baskunov B.P., Kochkina G.A., Ozerskaya S.M. (2012). Secondary Metabolite Profiles of the Penicillium Fungi Isolated from the Arctic and Antarctic Permafrost as Elements of Polyphase Taxonomy. Microbiology.

[88] Limame R., Wouters A., Pauwels B., Fransen E., Peeters M., Lardon F., De Wever O., Pauwels P. (2012). Comparative Analysis of Dynamic Cell Viability, Migration and Invasion Assessments by Novel Real-Time Technology and Classic Endpoint Assays. PLoS ONE.

[89] Zemskova M., Lilly M.B., Lin Y.W., Song J.H., Kraft A.S. (2010). p53-Dependent Induction of Prostate Cancer Cell Senescence by the PIM1 Protein Kinase. Mol. Cancer Res..

[90] Krishan A. (1975). Rapid flow cytofluorometric analysis of mammalian cell cycle by propidium iodide staining. J. Cell Biol..

[91] Jonkman J.E., Cathcart J.A., Xu F., Bartolini M.E., Amon J.E., Stevens K.M., Colarusso P. (2014). An introduction to the wound healing as say using live-cell microscopy. Cell Adh Migr..

[92] Livak K.J., Schmittgen T.D. (2001). Analysis of relative gene expression data using real-time quantitative PCR and the 2(-Delta Delta C(T)) Method. Methods.

[93] Yuan K., Kucik D., Singh R.K., Listinsky C.M., Listinsky J.J., Siegal G.P. (2008). Alterations in Human Breast Cancer Adhesion-Motility in Response to Changes in Cell Surface Glycoproteins Displaying α-L-Fucose Moieties. Int. J. Oncol..

