# Supplementary material for: Fumiquinazolines F and G from the Fungus Penicillium thymicola Demonstrates Anticancer Efficacy Against Triple-Negative Breast Cancer MDA-MB-231 Cells by Inhibiting Epithelial–Mesenchymal Transition
Source: Int J Mol Sci. 2025 Aug 5;26(15):7582. doi: 10.3390/ijms26157582 (PMC12347039; doi:10.3390/ijms26157582)
Supplement: Supplementary file 1 [file ijms-26-07582-s001.zip › ijms-3739159-supplementary.pdf]

Supplementary figures:

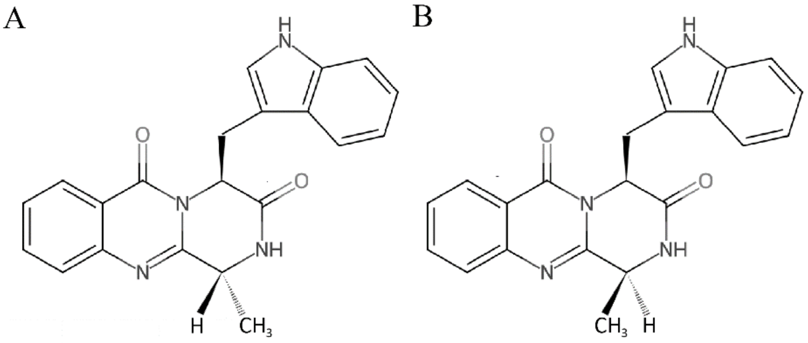

Figure S1. The structure of fumiquinazoline F (**A**) and fumiquinazoline G (**B**).

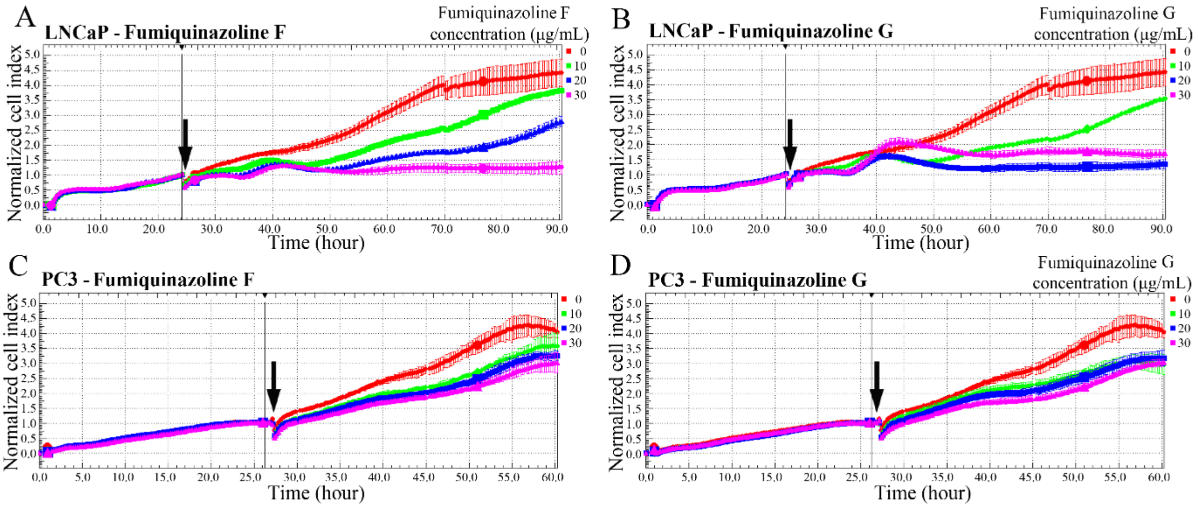

Figure S2. Fumiquinazolines F and G inhibit prostate cell growth. The hormone-dependent LNCaP cells (**A**,**B**) are sensitive, while hormone-independent PC3 cells are more resistant to the treatment (**C**,**D**). Monitoring of LNCaP and PC3 cell growth was tracked in real time using the xCELLigence technology. The X-axis represents the time of the treatment, and the Y-axis shows the cell index values (a parameter proportional to cell density). Black arrows on the graphs indicate the time at which fumiquinazolines were added. The graphs depict one example experiment from three separate studies.

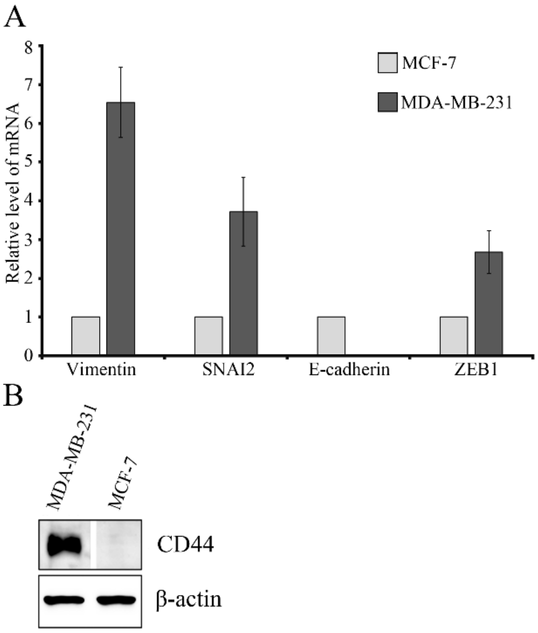

Figure S3. Expression of EMT markers in MCF-7 and MDA-MB-231 cells. (**A**) RT-qPCR analysis of mRNA expression in MDA-MB-231 vs. MCF-7 cells. The increased levels of EMT markers vimentin and transcriptional factors SNAI2 and ZEB1 are indicated, while expression of epithelial marker E-cadherin is low. Error bars represent ± standard deviation. The Y-axis shows mRNA expression change in MDA-MB-231 relative to the corresponding level in MCF-7 cells. The X-axis displays the tested EMT markers. (**B**) Western blot analysis of MDA-MB-231 and MCF-7 cell lysates. The high CD44 expression indicates the mesenchymal phenotype of triple-negative MDA-MB-231 cells, in contrast to epithelial MCF-7. β-actin was used as a control for protein loading.

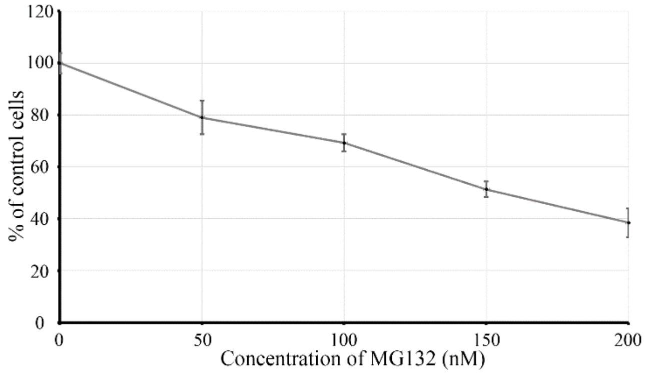

Figure S4. The proteasomal inhibitor MG 132 exhibits significant toxicity towards MDA-MB-231 breast cancer cells. Cells were cultured for 24 hours with varying doses of MG132 and subsequently assessed using crystal violet staining. The Y-axis represents the percentage of viable cells relative to the untreated control group. The X-axis represents MG 132 concentration in nanomoles.
